# Associations of monoamine oxidase A gene first exon methylation with sexual abuse and current depression in women

**DOI:** 10.1007/s00702-018-1875-3

**Published:** 2018-03-29

**Authors:** David Checknita, Tomas J. Ekström, Erika Comasco, Kent W. Nilsson, Jari Tiihonen, Sheilagh Hodgins

**Affiliations:** 1 Department of Neuroscience, Uppsala University, Uppsala, Sweden; 2 Department of Clinical Neuroscience, Karolinska Institute, Stockholm, Sweden; 3 Centre for Clinical Research, Västmanland County Council, Uppsala University, Västerås, Sweden; 4 Institut Universitaire en Santé Mentale de Montréal, Université de Montréal, Montreal, Canada; 5 Karolinska Universitetssjukhuset, Psychiatry Building R5:00 c/o Jari Tiihonen, 171 76 Stockholm, Sweden

**Keywords:** Child abuse, Depression, Epigenetics, Gene–environment interaction methylation, Women

## Abstract

Childhood physical abuse (PA) and sexual abuse (SA) interact with monoamine oxidase A (*MAOA*) gene polymorphism to modify risk for mental disorders. In addition, PA and SA may alter gene activity through epigenetic mechanisms such as DNA methylation, thereby further modifying risk for disorders. We investigated whether methylation in a region spanning the *MAOA* first exon and part of the first intron was associated with PA and/or SA, *MAOA* genotype, alcohol dependence, drug dependence, depression disorders, anxiety disorders, and conduct disorder. 114 Swedish women completed standardized diagnostic interviews and questionnaires to report PA and SA, and provided saliva samples for DNA extraction. DNA was genotyped for MAOA-uVNTR polymorphisms, and methylation of a *MAOA* region of interest (chrX: 43,515,544–43,515,991) was measured. SA, not PA, was associated with hypermethylation of the *MAOA* first exon relative to no-abuse, and the association was robust to adjustment for psychoactive medication, alcohol and drug dependence, and current substance use. SA and MAOA-uVNTR genotype, but not their interaction, was associated with *MAOA* methylation. SA associated with all measured mental disorders. Hypermethylation of *MAOA* first exon mediated the association of SA with current depression, and both methylation levels and SA independently predicted lifetime depression. Much remains to be learned about the independent effects of SA and MAOA-uVNTR genotypes on methylation of the *MAOA* first exon.

**Electronic supplementary material:**

The online version of this article (10.1007/s00702-018-1875-3) contains supplementary material, which is available to authorized users.

## Introduction

The prevalence of childhood physical abuse (PA) and sexual abuse (SA) in the US is estimated at 28.4 and 4.5%, respectively (Hussey et al. 2006). Smaller proportions of girls than boys experience PA (Thompson et al. 2004) and greater proportions experience SA (Singh et al. 2014). Meta-analyses indicate that childhood PA and SA are associated with greatly increased risks for conduct disorder (CD) in childhood, antisocial personality disorder (ASPD) in adulthood, and substance use, depression, and anxiety disorders (Hussey et al. 2006; Springer et al. 2007; Fergusson et al. 2008; Singh et al. 2014; Li et al. 2016; Rita et al. 2016); all disorders associated with serotonergic dysfunction (Comai et al. 2012; Booij et al. 2015). Both PA and SA are associated with abnormalities of gray and white matter brain structures (McCrory et al. 2010), and activity (Herringa et al. 2013; Teicher and Samson 2016), particularly in limbic and hippocampal structures which are targets of serotonergic projections. Furthermore, childhood maltreatment modifies the association of genetic polymorphisms involved in the regulation of brain serotonin with antisocial behaviour (Pavlov et al. 2012) and depression (Mandelli and Serretti 2013).

Within the promoter region of the *MAOA* gene, which encodes the monoamine oxidase (MAO) enzyme that metabolizes serotonin and other monoamine neurotransmitters, there is a 30 base-pair functional variable number of tandem repeats polymorphism (MAOA-uVNTR) including short (MAOA-S) and long (MAOA-L) variants associated with low expression and high expression, respectively, in cell cultures (Sabol et al. 1998; Beach et al. 2010).

The expression of *MAOA* may be altered by environmental factors through epigenetic mechanisms. A region of interest (ROI) within the *MAOA* promoter spanning the first exonic and intronic regions was previously identified and characterized as a putative locus for methylation (Shumay and Fowler 2010). Methylation of this region was negatively correlated with brain MAO enzymatic activity in healthy men (Shumay et al. 2012). Male offenders with ASPD showed hypermethylation in this region relative to healthy men, which was associated with whole-blood serotonin levels among offenders and reduced transcriptional activity in vitro (Checknita et al. 2015). However, it is not known if hypermethylation is specifically associated with ASPD or with maltreatment that is common among men with ASPD (Hill and Nathan 2008), or substance use disorders that characterize almost all men with ASPD (Goldstein et al. 2017), or anxiety disorders presented by approximately half of male offenders with ASPD (Hodgins et al. 2010), or depression that is also elevated in this population (Lenzenweger et al. 2007). Hypomethylation of this region was observed among women with depression (Melas et al. 2013; Melas and Forsell 2015) and those with panic disorder relative to healthy women (Ziegler et al. 2016). Stressful life events similarly associate with depression and anxiety (McCrory et al. 2012). Hypermethylation of *MAOA* exon 1 was also observed among women relative to men, suggestive of X-chromosome inactivation given the gene’s location on the X-chromosome (Melas and Forsell 2015). It is not presently known whether PA and SA are associated with *MAOA* promoter methylation.

Differentially methylated regions in candidate genes associated with mental disorders are often located in regions flanking transcription start sites and typically do not overlap with known functional polymorphisms in the same gene promoters (Dammann et al. 2011; Checknita et al. 2015). It is presently not known whether genotype and interactions of genotype and environmental factors are associated with altered methylation. One study showed that lower promoter methylation levels of the serotonin transporter gene (*5-HTT*) were associated with cortisol responses to a stress test only among carriers of the low-expressing variant of the 5HTTLPR promoter polymorphism (5HTT-S) (Alexander et al. 2014). Among adult carriers of 5HTT-S, childhood PA was associated with reduced *5HTT* mRNA levels, though methylation was not involved (Alexander et al. 2014). It is not currently known if *MAOA* genotype interacts with childhood maltreatment to determine methylation levels in the *MAOA* ROI.

Additional evidence that environmental factors lead to epigenetic changes, and that the magnitude of the changes depends on genotype, would have implications for treatment. For example, among women with panic disorder who responded positively to cognitive-behavioural therapy, methylation levels rose to equal those observed among healthy women, while no significant change in methylation was detected among treatment non-responders (Ziegler et al. 2016). Although the role of MAOA-uVNTR genotype in treatment response is not fully understood, recent studies suggest that men and women carrying MAOA-S, as compared to MAOA-L carriers, display greater reductions in symptoms of depression when taking mirtazapine (Tzeng et al. 2009) and of panic disorder following cognitive-behavioural treatment (Reif et al. 2014).

The present study examined methylation of an ROI within the *MAOA* gene promoter flanking the first exonic region in DNA derived from saliva samples of young women, most of whom had displayed antisocial behaviour in adolescence, and one-half of whom had experienced PA and/or SA. The first aim was to determine whether PA and/or SA were associated with methylation in the *MAOA* ROI, and whether any observed associations were due to lifetime or current psychoactive medication, lifetime or current alcohol or drug dependence, current alcohol or drug use, each of which is common among persons who have experienced PA and/or SA, each of which has been shown to be associated with methylation levels in *MAOA* ROI and/or wide-spread epigenetic alterations across the genome (Philibert et al. 2008; Nestler 2014; Booij et al. 2015; Szyf 2015; Ziegler et al. 2016). The second aim was to determine whether associations between abuse and methylation differed by *MAOA* genotype. The third aim was to determine whether methylation of the *MAOA* ROI mediates associations of abuse with alcohol dependence, drug dependence, depression disorders, anxiety disorders, and CD.

## Method

### Participants

The sample included 114 Swedish women aged on average 22 years, 91 were recruited at a clinic for adolescent substance misuse, 75 of whom sought treatment and 16 of whom were non-biological siblings of other clinic attendees (not biologically related to any other participant), and 23 healthy women. The 75 ex-clients completed structured, validated, diagnostic interviews and questionnaires to report on PA and SA at first contact with the clinic and 6, 12, 60, and 75 months later. At the 60-month follow-up, the 16 non-biologically related siblings completed the same assessments, and all 91 participants provided saliva samples for DNA extraction. At the 75-month follow-up, the healthy female participants matched on age to other participants were recruited, completed assessments similar to those completed by the other participants, and provided saliva for DNA extraction.

### Measures

#### DNA

Genomic DNA was extracted with a standard in-silica-based method from saliva samples collected with the Oragene Self-Collection Kit (DNA Genotek Inc. Ottawa, ON, Canada) according to the manufacturer’s guidelines. Saliva samples were collected during the meeting between the participant and the researchers, using the Oragene self-collection kit (DNA Genotek^®^, Canada: http://www.dnagenotek.com). The samples were stored at room temperature in accordance with the manufacturer’s guidelines. Genomic DNA was extracted from 200 µl of saliva using the silica-based Kleargene DNA extraction method (LGC^®^, UK: http://www.lgcgroup.com) and stored at − 20 °C, and then − 80 °C, prior to genotyping procedures and methylation analysis of the *MAOA* ROI. Genotyping and methylation analyses were performed in a blinded manner.

#### Genotypes

Genotyping was performed using a standard PCR technique, followed by gel electrophoresis. The target 30-bp repeat target-region of *MAOA* (MAOA-uVNTR) was amplified using forward primer 5′ ACA GCC TGA CCG TGG AGA AG 3′ and reverse primer 5′ GAA CGG ACG CTC CAT TCG GA 3′ (Sabol et al. 1998). In accordance with prior in vitro functional analyses of the MAOA-uVNTR (Beach et al. 2010), the 3 repeat variant was defined as the short (*MAOA*-S) allele, and 3.5, 4, or 5 repeat variants as the long (*MAOA*-L) allele. Genotype prevalence is presented in Table 1. Hardy–Weinberg Equilibrium was verified for MAOA-uVNTR genotype using an *X*^*2*^ test (*p* = 0.47). Allelic frequencies of MAOA-uVNTR in our study were consistent with the results of previous studies of Swedish females (Åslund et al. 2013).

**Table 1:** Characteristics of participants carrying different *MAOA* genotypes

	MAOA-uVNTR^a^ (*n* = 114)			Statistics
	SS	SL	LL	
Participants	*n* = 20	*n* = 51	*n* = 43	
% Participants	17.5	44.7	37.7	
Mean (SD) age at saliva collection	22.56 (3.95)	22.00 (3.31)	22.38 (3.13)	ns
Physical and sexual abuse				
% (*n* =) physical and/or sexual abuse	45.0 (9)	64.7 (33)	53.5 (23)	ns
% (*n* =) physical abuse only	8.3 (1)	18.2 (4)	16.7 (4)	ns
% (*n* =) sexual abuse only	21.4 (3)	43.8 (14)	31.0 (9)	ns
Lifetime diagnoses				
% (*n* =) alcohol dependence	10.0 (2)	33.3 (17)	25.6 (11)	ns
% (*n* =) drug dependence	25.0 (5)	23.5 (12)	30.2 (13)	ns
% (*n* =) anxiety disorders	40.0 (8)	68.6 (35)	51.2 (22)	ns
% (*n* =) depression disorders	55.0 (11)	68.6 (35)	51.2 (26)	ns
% (*n* =) conduct disorder before 15	30.0 (6)	41.2 (21)	37.2 (16)	ns
Current diagnoses				
% (*n* =) alcohol dependence	5.0 (1)	19.6 (10)	14.0 (6)	ns
% (*n* =) drug dependence	5.0 (1)	11.8 (6)	20.9 (9)	ns

	MAOA-uVNTR^a^ (*n* = 143)			
	SS	SL	LL	
% (*n* =) anxiety disorders	20.0 (4)	62.7 (32)	44.2 (19)	*χ*^2^(2, *N* = 114) = 10.97 *p* = 0.004
% (*n* =) depression disorders	45.0 (9)	43.1 (22)	32.6 (14)	ns

*SD* standard deviation, *LL* homozygous for the long alleles, *SL* heterozygous, *SS* homozygous for the short alleles, *ns* not significant

^a^Monoamine oxidase A variable number of tandem repeats

#### Methylation analysis of *MAOA* promoter region of interest

Methylation analysis targeted a previously characterized 448-bp region of interest (hg19 chrX: 43,515,544–43,515,991) within the *MAOA* promoter comprised of 16 CpGs spanning the first exon and part of the first intronic region (Checknita et al. 2015). Genomic DNA extracted from saliva was first bisulfite-treated using EZ DNA Methylation™ Kit (Zymo Research Corporation, Irvine, CA) and then assayed using Agena Bioscience’s EpiTYPER at Karolinska University Hospital Mutation Analysis Core Facility (MAF). Resulting data represented the percentage of methylation at each CpG to the nearest 0.5%. CpGs were denoted numerically based on their 5′–3′ position within the ROI based on the forward strand genomic sequence. To ensure optimal technical outcomes, an amplicon designed on the reverse strand covered CpGs 1–13 and another amplicon covering CpGs 13–16 was designed on the forward strand. For additional information regarding the EpiTYPER procedure and quality control, please refer to Online Resource 1.

#### Physical abuse

Participants completed the revised Conflict Tactics Scales (Straus et al. 1996) to report on physical abuse (PA) by parents. Responses were dichotomously coded as absent or present (hit with fist or kicked hard, hit on part of body other than bottom with hard object, thrown or knocked down, grabbed around neck and choked, hit repeatedly very hard, burned, or threatened with a gun or knife).

#### Sexual abuse

Sexual abuse (SA) was assessed using items from the Sexual Experience Survey (Koss and Oros 1982), the Sexual and Physical Abuse Questionnaire (Kooiman et al. 2002), and McArthur Community Violence Instrument (Steadman et al. 1998). SA was dichotomously coded as absent or present if any of the following were reported: forced to have sex against her will by a person in authority, by offering alcohol or drugs, or by physical violence.

#### Lifetime and current mental disorders

All participants completed the Structured Clinical Interview for DSM-IV, both I and II (SCID I, SCID II) at DNA collection. Lifetime and current (past 6 months) diagnoses of alcohol dependence, drug dependence, depression disorder (major depression disorder, dysthymia, depression disorder not-otherwise-specified, or substance-induced mood disorder), anxiety disorder (agoraphobia, generalized anxiety disorder, anxiety disorder not-otherwise-specified, obsessive compulsive disorder, panic disorder, post-traumatic stress disorder, social phobia, specific phobia, or substance-induced anxiety disorder), and conduct disorder (CD), prior to age 15, were recorded. Thus, CD and lifetime diagnoses were made prior to DNA collection (baseline, 6-, 12-, and 60-months follow-ups) for 91 participants and reconfirmed at the time of the DNA collection when the current diagnoses were made. For the other 23 participants, lifetime and current diagnoses were made when DNA was collected.

#### Current alcohol and drug use

At the time of DNA collection, participants completed the 10-item AUDIT questionnaire (Babor et al. 2001) reporting number of drinks consumed, frequency of drinking, frequency of heavy drinking, alcohol dependence symptoms, and alcohol-related problems during the past 12 months. Responses were summed to obtain a total score ranging from 0 to 40. A higher score indicated more alcohol-related problems. In addition, at the time of DNA collection, participants completed the 11-item DUDIT questionnaire (Berman et al. 2005) on report frequency of drug use, frequency of heavy drug use, polydrug use, drug dependence symptoms, and drug-related problems during the past 12 months. Responses were summed to obtain a score ranging from 0 to 44. A higher score indicated more drug-related problems.

#### Psychoactive medication use

At all waves of data collection, participants reported use of psychoactive medications including stimulants, hypnotics, anxiolytics, antidepressants, antipsychotics. Lifetime and current (past 6 months) use of psychoactive medication was coded present or absent.

#### Statistical analyses

Chi-square and one-way ANOVAs were computed to compare characteristics of carriers of MAOA-uVNTR genotypes. To explore associations between abuse and *MAOA* ROI methylation levels, two-way mixed-model ANOVAs with post hoc LSD for multiple comparisons were computed. These models were used to test group differences in mean overall ROI methylation and at each CpG comparing: (1) 65 participants who had experienced PA and/or SA to 49 non-abused (NA) participants who experienced neither PA nor SA. Power analyses were calculated for each of the three comparisons using average group differences in ROI methylation (mean 2.56, SD 4.27) from (Checknita et al. 2015): PA and/or SA versus NA included 114 participants and provided 0.97 power to detect a similar group difference at an alpha of 0.05; PA only versus NA included 58 participants providing 0.78 power; and SA versus NA included 75 participants with 0.87 power. One-way ANCOVAs were computed to determine if associations between maltreatment and *MAOA* ROI methylation were robust to adjustments for lifetime and current use of psychoactive medications (stimulants, hypnotics, anxiolytics, antidepressants, and antipsychotics), lifetime and current diagnoses of alcohol dependence, current alcohol use, lifetime and current diagnoses of drug dependence, and current drug use.

Next, general-linear regression models (GLMs) were computed to examine associations of *MAOA* genotype, abuse, and interactions of genotype and abuse with methylation at each locus and mean methylation of exon 1. The model included three predictors: *MAOA* genotype, SA, and the interaction of *MAOA* genotype and SA.

Chi-square analyses were used to examine associations of abuse with lifetime and current diagnoses of mental disorders. Step-wise logistic regression models were computed to determine whether methylation levels at each locus mediated associations between abuse and diagnoses. Abuse was entered into step 1; methylation was entered in step 2; and in step 3, both abuse and methylation were entered. If abuse was significantly associated with a diagnosis in step 1, abuse was significantly associated with methylation in step 2, and only methylation was associated in step 3, results were interpreted to suggest that methylation fully mediated the association between abuse and the clinical diagnosis. To verify if mediation effects of methylation differed significantly from the direct associations of SA and diagnoses, PROCESS for SPSS v2.15 was used with the bootstrapping procedure for indirect effects described by Hayes (2013).

## Results

As presented in Table 1, neither age nor proportions of participants with PA and/or SA, PA, SA, lifetime diagnoses, or CD before age 15 varied by *MAOA* genotype. Proportionately, more of the *MAOA*-SL carriers, than SS or LL, presented, at a trend, lifetime diagnoses of anxiety disorders. Current diagnoses varied by genotype: proportionately more MAOA-SL carriers, than SS or LL, presented anxiety disorders. Characteristics of participants that were entered into analyses as covariates are presented on Online Resource Table S1.

### Are physical and sexual abuse associated with *MAOA* promoter ROI methylation?

#### PA and/or SA

A two-way mixed-model ANOVA revealed a significant main effect of group [*F*(1, 204) = 13.622, *p* < 0.001] indicating higher methylation among those who had experienced PA and/or SA compared to NA. Post hoc LSD analysis revealed significantly higher methylation levels among those who experienced PA and/or SA compared to NA at CpG sites 2/3 (*p* < 0.001), 4 (*p* < 0.001), 5/6 (*p* < 0.001), 7/8 (*p* < 0.001), 10 (*p* < 0.001), and 11 (*p* = 0.002). Lower methylation among those who experienced PA and/or SA compared to NA was found at CpG15 (*p* = 0.036). One-way ANCOVAs indicated that these associations were robust to adjustments for lifetime and current use of psychoactive medications, lifetime and current diagnosis of alcohol dependence and current alcohol use, and lifetime and current drug dependence and current drug use.

#### PA only

A two-way ANOVA revealed a significant main effect of group [*F*(1, 115) = 4.951, *p* = 0.028] indicating higher methylation among those who had experienced PA compared to NA. Post hoc LSD analysis revealed significantly higher methylation levels among those who had experienced PA at CpG site 7/8 (*p* = 0.023). Only nine participants experienced PA only. Therefore, these results were not adjusted for covariates.

#### SA only

**Fig. 1:**
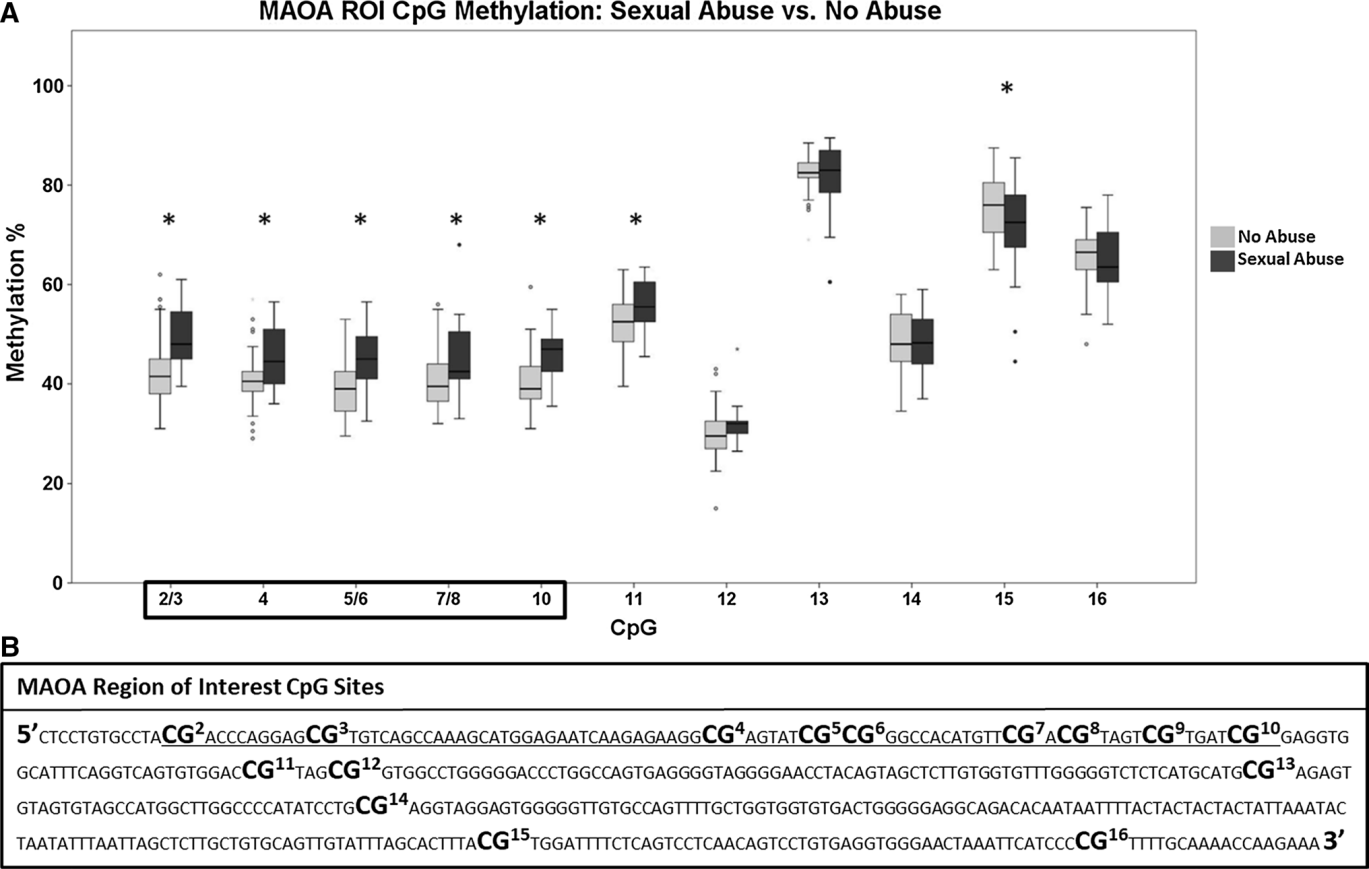
**a** Results of comparisons of methylation levels across the *MAOA* ROI CpGs (*p < 0.05) observed among women who had and who had not experienced sexual abuse. No data were available for CpGs 1 and 9. **b** Genomic sequence of the *MAOA* ROI with all CpG sites enlarged, bolded, and numbered, and with the exonic protein coding region sequence underlined

A two-way ANOVA revealed a significant main effect of group [*F*(1, 187) = 12.693, *p* < 0.001] indicating higher overall ROI methylation levels among participants who had experienced SA compared to those with NA. Post hoc LSD revealed significantly higher methylation levels among the SA group compared to NA which were again specific to *MAOA* first exon CpG sites 2/3 (*p* < 0.001), 4 (*p* = 0.001), 5/6 (*p* < 0.001), 7/8 (*p* = 0.001), and 11 (*p* = 0.002). Lower methylation among those who experienced SA only compared to NA was found at CpG15 (*p* = 0.018). Results are illustrated in Fig. 1. One-way ANCOVAs revealed that these associations remained robust after adjusting for all covariates.

### Does sexual abuse interact with *MAOA* genotypes to modify the association between sexual abuse and *MAOA* first exon CpG and mean methylation?

Table 2 presents the results from regression models predicting exon 1 mean methylation and methylation at CpG 2/3, 4, 5/6, 7/8, 10, 11, and 15 that included three predictors: *MAOA* genotype, SA, and the interaction of *MAOA* genotype and SA. Results indicated that genotype was predictive of methylation levels at CpGs 2/3, 4, 10, and mean exon 1 methylation levels. SA was predictive of methylation levels at CpG 2/3, 4, 5/6, 10, and mean exon 1 methylation levels.

**Table 2:** Results of regression models predicting *MAOA* Exon 1 methylation by *MAOA* genotype and sexual abuse

	General-linear model			Observed power
	*df*	*F*	*p*	
CpG2/3				
MAOA-uVNTR^a^	1	7.168	0.028	0.384
Sexual abuse	1	16.173	< 0.001	0.967
MAOA-uVNTR × sexual abuse		1.752	0.416	0.182
Adjusted *R*^2^ = 0.186				
CpG4				
MAOA-uVNTR	1	6.161	0.046	0.087
Sexual abuse	1	13.015	<0.001	0.926
MAOA-uVNTR × sexual abuse		2.716	0.257	0.263
Adjusted *R*^2^ = 0.146				
CpG5/6				
MAOA-uVNTR	1	4.507	0.105	0.131
Sexual abuse	1	17.029	<0.001	0.974
MAOA-uVNTR × sexual abuse		2.856	0.240	0.275
Adjusted *R*^2^ = 0.197				
CpG7/8				
MAOA-uVNTR	1	1.888	0.389	0.192
Sexual abuse	1	8.522	0.004	0.788
MAOA-uVNTR × sexual abuse		0.506	0.776	0.085
Adjusted *R*^2^ = 0.068				
CpG10				
MAOA-uVNTR	1	10.583	0.005	0.546
Sexual abuse	1	16.276	<0.001	0.968
MAOA-uVNTR × sexual abuse		0.412	0.814	0.078
Adjusted *R*^2^ = 0.206				
Exon 1 mean methylation				
MAOA-uVNTR	1	7.188	0.027	0.262
Sexual abuse	1	20.097	<0.001	0.989
MAOA-uVNTR × sexual abuse		1.223	0.542	0.139
Adjusted *R*^2^ = 0.214				
CpG11				
MAOA-uVNTR	1	4.662	0.097	0.306
Sexual abuse	1	6.452	0.011	0.670
MAOA-uVNTR × sexual abuse		0.018	0.991	0.051
Adjusted *R*^2^ = 0.072				
CpG15				
MAOA-uVNTR	1	1.108	0.575	0.058
Sexual abuse	1	1.971	0.160	0.264
MAOA-uVNTR × sexual abuse		0.936	0.626	0.117
Adjusted *R*^2^ = 0.004				

^a^Monoamine oxidase A variable number of tandem repeats

### Does *MAOA* first exon methylation mediate associations of sexual abuse with alcohol dependence, drug dependence, depression disorders, anxiety disorders, and CD?

Significantly greater proportions of participants who experienced SA than NA presented both lifetime and current diagnoses of alcohol dependence, drug dependence, anxiety disorders, and CD prior to age 15 (see Online Resource Table S2). In addition, a greater proportion of participants who experienced SA than of the non-abused presented lifetime depressive disorders, while the proportions with the current depression were similar.

Results of significant final step-wise models testing whether methylation mediated associations of SA with diagnoses are presented in Table 3 and all results in Online Resource Tables S3 and S4. Mediation was detected only for increased risk of current depression. Methylation at CpGs 2/3, 4, 5/6, 7/8, and exon 1 mean levels mediated the association between SA and current depression. As presented in Table 3, methylation at CpGs 2/3, 4, 5/6, 7/8, and of exon 1 fully mediated the association of SA and depression. Lifetime depressive disorders were predicted independently by SA and methylation at CpG 5/6. By contrast, lifetime alcohol dependence was predicted only by SA.

**Table 3:** Factors associated with current and lifetime disorders

Predictor	Step 1		Step 2		Step 3		Mediation analysis^a^
	Odds ratio	95% confidence interval	Odds ratio	95% confidence interval	Odds ratio	95% confidence interval	Bootstrapped 95% confidence interval
Lifetime depression disorders							
Sexual abuse	5.60	1.95–16.04			3.37	1.08–10.54	
CpG5/6			1.14	1.05–1.24	1.10	1.01–1.21	
					*χ*^2^(1, *N* = 75) = 15.30, *p* < 0.001		–
Current depression disorders							
Sexual abuse	5.60	1.95–16.04			1.20	0.38–3.80	
CpG2/3			1.11	1.03–1.20	1,11	1.02–1.20	
					*χ*^2^(1, *N* = 75) = 8.77, *p* = 0.012		0.13–1.33
Sexual abuse	5.60	1.95–16.04			1.38	0.44–4.26	
CpG4			1.11	1.02–1.21	1.10	1.00–1.21	
					*χ*^2^(1, *N* = 75) = 6.05, *p* = 0.049		1.33–1.42
Sexual abuse	5.60	1.95–16.04			1.01	0.30–3.35	
CpG5/6			1.14	1.04–1.25	1.14	1.04–1.26	
					*χ*^2^(1, *N* = 75) = 9.96, *p* = 0.007		0.16–2.01
Sexual abuse	5.60	1.95–16.04			1.40	0.46–4.29	
CpG7/8			1.11	1.02–1.20	1.10	1.01–1.19	
					*χ*^2^(1, *N* = 75) = 7.10, *p* = 0.029		0.04–1.22
Sexual abuse	5.60	1.95–16.04			1.02	0.31–3.39	
Exon 1			1.16	1.05–1.28	1.16	1.04–1.29	
					*χ*^2^(1, *N* = 75) = 9.74, *p* = 0.008		0.17–1.22

^a^Mediation analysis conducted using bootstrapped bias-corrected 95% confidence intervals with 5000 bootstrapping samples

### Post hoc analyses

Women with current depression (*n* = 46) as compared to women with no lifetime or current depression (*n* = 45) presented higher levels of methylation at CpGs, 2/3 [*t*(88) = 2.63, *p* = 0.010], 5/6 [*t*(88) = 2.98, *p* = 0.004], 7/8 [*t*(88) = 2.70, *p* = 0.008], 10 [*t*(88) = 2.04, *p* = 0.004], 11 [*t*(88) = 1.99, *p* = 0.050], and 12 [*t*(88) = 2.00, *p* = 0.049], as well as mean methylation levels of the first exon [*t*(88) = 2.81, *p* = 0.006] and ROI [*t*(88) = 3.23, *p* = 0.002]. Among the women with current depression, a higher level of methylation was associated with past or current use of any medication (stimulants, hypnotics, anxiolytics, antidepressants, and antipsychotics) at CpG 7/8 [*t*(34) = 2.04, *p* = 0.049]. No differences in methylation were observed when antidepressants were considered alone. Women with current or lifetime diagnoses of panic disorder (*n* = 36) as compared to women with no current or lifetime diagnoses of anxiety or depression (*n* = 48) presented higher levels of methylation of the first exon [*t*(70) = 3.43, *p* = 0.001] and ROI [*t*(70) = 3.33, *p* = 0.001], as well as at CpGs 2/3 [*t*(70) = 3.64, *p* = 0.001], 4 [*t*(70) = 2.10, *p* = 0.040], 5/6 [*t*(70) = 3.65, *p* = 0.001], 7/8 [*t*(70) = 2.29, *p* = 0.025], and 10 [*t*(70) = 2.88, *p* = 0.005].

## Discussion

Among young women, SA was associated with hypermethylation of the *MAOA* ROI and particularly the first exonic region of *MAOA*. This association was robust to adjustments for factors common among individuals who have experienced maltreatment, including psychoactive medication, alcohol and drug dependence, and current use of alcohol and drugs. The first exonic region contains binding domains for transcription factors associated with transcriptional enhancement (See Online Resource Table S5). First exon methylation of genes is suggested to play a stronger role in transcriptional silencing through the blockade of transcript initiation, compared to methylation in upstream promoter loci which contributes to up or downregulation of transcriptional activity (Brenet et al. 2011). Methylation of the region flanking the *MAOA* first exon has been associated with MAO enzymatic activity in the brain (Shumay et al. 2012), transcriptional downregulation in vitro, and with 5-HT levels in whole blood (Checknita et al. 2015). In a recent study, higher methylation levels specific to the *MAOA* first exon were observed in women (mean 45.3%) compared to men (mean 13.7%) (Melas and Forsell 2015). Here, we report a similar level of methylation at *MAOA* exon 1 (mean 44.3%, SD 5.9) in women. Thus, the higher levels of *MAOA* first exon methylation observed among women are indicative of sites involved in X-chromosome inactivation of one of the two *MAOA* alleles by DNA methylation (Cotton et al. 2011), further suggesting a functional role of methylation in this region. While the study focused primarily on the well-characterized first exonic region of *MAOA*, it is noteworthy that SA was also associated with hypomethylation of intronic CpG 15, ~ 260-bp downstream from the first exonic region. Similar to increased exonic methylation, lower intronic methylation also typically associates with transcriptional downregulation (Jones 2012; Moore et al. 2013). As such, altered intronic methylation of *MAOA* among those who experienced SA may have also contributed to the associations reported here.

While SA was consistently associated with hypermethylation of the *MAOA* ROI, similar results regarding PA were less clear due to the small number of participants who experienced PA only. The previous studies of methylation in this region among females also reported no association with a measure of adversity defined as parental death, divorce, financial problems, or other family problems (Melas et al. 2013; Melas and Forsell 2015).

First exon methylation was predicted by *MAOA* genotype and by SA, but not by the interaction of genotype and SA. These findings are consistent with a prior study that showed no association of *MAOA* genotype with *MAOA* methylation or brain MAO enzymatic activity in men (Shumay et al. 2012). Another study showed that reductions of panic disorder symptoms following cognitive-behavioural treatment were accompanied by *MAOA* exon 1 methylation levels similar to those of healthy women and that changes in methylation levels did not differ by genotype (Ziegler et al. 2016). MAOA-SS is associated with positive response to an antidepressant, mirtazapine in men (Tzeng et al. 2009), and to cognitive-behavioural therapy in men and women (Reif et al. 2014), and amygdala hyperreactivity in men (Meyer-Lindenberg et al. 2006) suggesting increased sensitivity to environmental factors. Yet, in the present study, *MAOA* genotype did not influence the effect of SA on methylation levels. Deficient expression of *MAOA* occurs early in life leading to inhibited outgrowth of the serotonergic system and low basal serotonin levels (Nordquist and Oreland 2010; Booij et al. 2015), that when impacted by negative environmental factors later in life promote mental disorders (Byrd and Manuck 2014). The results of the present study, while preliminary and in need of replication, suggest that this effect occurs independently of methylation.

*MAOA* exon 1 hypermethylation was shown to mediate associations between SA and current depression. In addition, SA and hypermethylation of the *MAOA* ROI were independently associated with lifetime depression. Although the observed mediation effects were small, this finding is consistent with the notion that many biological and environmental factors are involved in modulating associations between SA and depression (Labonte and Turecki 2012).

While the present study consistently showed that hypermethylation of the *MAOA* ROI was associated with depression, two previous studies of males and females reported hypomethylation in this same region among depressed women (Melas et al. 2013; Melas and Forsell 2015). The two previous studies included small samples with wide age ranges, a different measure of adversity, no measure of genotypes, and participants that may differ in comorbid disorders from the present sample. In the present study, almost all of the women with current depression had a long history of depression, and 37.7% had presented CD prior to age 15. Depression is commonly comorbid with CD in adolescence (Angold et al. 1999), and adolescent CD predicts severe depression in adult women (Choi et al. 2016). We found that SA was directly associated with CD, depression, and hypermethylation of the *MAOA* ROI. Given the previous evidence of hypermethylation of this same region among male offenders with ASPD, it may be that hypermethylation of this region of *MAOA* is associated specifically with depression among females with prior antisocial behaviour and experiences of SA, consistent with the notion that both the presentation and etiology of depression are heterogeneous (Januar et al. 2015).

Unfortunately, many young females experience SA, which as the results of the present study highlight, is associated with alcohol dependence, drug dependence, anxiety, depression, and CD. The present study shows that SA was associated with hypermethylation of *MAOA* exon 1. Furthermore, hypermethylation of this region mediated the association of SA with current depression and was independently associated with lifetime depression. Elucidating neurobiological mechanisms by which SA promotes the development of mental disorders will provide a basis for the development of pharmacological and cognitive-behavioural interventions to limit the negative consequences of SA. The finding that reductions in symptoms of panic disorder following cognitive-behavioural treatment (Ziegler et al. 2016) were associated with a normalization of methylation levels underlines the importance of understanding the links between mental disorders and epigenetic mechanisms.

Robust evidence shows that *MAOA* genotype moderates the association between maltreatment and mental disorders (Wermter et al. 2010; Mandelli and Serretti 2013; Byrd and Manuck 2014), and recent evidence shows associations between *MAOA* methylation and mental disorders (Melas et al. 2013; Melas and Forsell 2015; Checknita et al. 2015; Ziegler et al. 2016). Furthermore, our study, and others (Melas et al. 2013; Melas and Forsell 2015; Ziegler et al. 2016), showed that genotype and environmental trauma independently associate with *MAOA* ROI methylation. Such findings, however, are preliminary and require replication. *MAOA* genotype acts early in life to regulate, or set, lifelong levels of serotonergic functioning (Nordquist and Oreland 2010). The level of serotonergic functioning may increase risk of altered methylation, though our results suggest that it does so independently of environmental trauma. By contrast, environmental trauma, such as SA, altered methylation regardless of *MAOA* genotype. Given that methylation levels were more strongly associated with SA than with genotype in our study, it may be that proximal traumas have the greatest impact on methylation. It may be of importance that *MAOA* methylation only mediated associations between SA and current depression, and not those with other disorders. This finding highlights the need for future studies exploring genotypes, epigenetics, environmental traumas, mental disorders, and the associations of various mental disorders with the interactions of these factors (Holz et al. 2014).

## Limitations and strengths

As this was a preliminary and exploratory study with a relatively small sample, we lacked sufficient statistical power to perform multiple corrections for all of the statistical models used, and particularly for the regression models that included only participants with SA and two predictors. However, the initial group comparisons of methylation of maltreated and non-maltreated women used Fisher’s LSD post hoc analyses with similar results obtained following more stringent Bonferroni corrections. These initial group comparisons used two-way mixed-model ANOVA models which included CpG as a repeated measure, thus allowing for the detection of group differences in overall methylation, and differences at each CpG corrected for multiple comparisons. As such, this approach offers a means to mitigate issues with multiple testing and has been adopted in the previous epigenetic studies (Labonte et al. 2012; Gross et al. 2013; Cruceanu et al. 2016).

The study design did not allow us to determine when alterations to methylation occurred, nor if it they were a direct consequence of maltreatment. Although our findings are in line with a large body of evidence associating maltreatment with altered methylation (Szyf 2011; Nagy and Turecki 2012), future prospective studies with repeated assessments of maltreatment and methylation are needed to determine if the association is direct and when the methylation changes occur. Type, severity, and chronicity of the maltreatment likely influence both the association and timing of the methylation changes (Szyf 2011; Nagy and Turecki 2012; Vachon et al. 2015). However, as summarized in Online Resource 1, analyses showed that women who had experienced one or both types of abuse exhibited increased exonic CpGs 1–10 and CpG 11 methylation levels relative to those who experienced no-abuse, with no differences between those who experienced one, or both, types of abuse at these same CpGs. No group differences in methylation for CpGs 12–16 were observed. These findings emphasize the importance of considering severity of abuse in future studies, particularly given that child victims often experience multiple types of abuse (Vachon et al. 2015). Few participants experienced PA only, thus limiting confidence in our results examining the association of PA only with methylation. In addition, data on nicotine use were not available. Smoking has been associated with altered methylation levels of *MAOA* in healthy participants (Philibert et al. 2010), but two previous studies of clinical samples of women indicated no associations between smoking and methylation levels in the *MAOA* ROI (Melas et al. 2013; Melas and Forsell 2015).

DNA was extracted from saliva which contains a diverse range of peripheral cell-types, thus limiting the interpretation of our results in relation to central nervous system processes. However, a previous study identified an inverse correlation between *MAOA* promoter methylation in whole-blood DNA and MAO enzymatic activity in the brain (Shumay et al. 2012). In addition, methylation patterns observed in our study are consistent with methylation patterns in the *MAOA* ROI reported elsewhere in whole-blood samples in women (Melas et al. 2013; Melas and Forsell 2015; Ziegler et al. 2016). Thus, measurements of *MAOA* methylation in peripheral tissues may be viable proxies for central processes. Furthermore, as the EpiTYPER technique cannot distinguish methylation between X-chromosome alleles, we were unable to examine X-chromosome inactivation. Given the potential importance of X-chromosome inactivation in the regulation of MAOA in women, its impact will be important to elucidate in subsequent epigenetic studies examining associations of abuse and mental disorders.

The study is also characterized by strengths including extensive data on mental disorders, antisocial behaviour, and maltreatment that were assessed with validated instruments. These rich data allowed us to determine that the observed association between SA and *MAOA* first exon methylation levels were robust to adjustment for lifetime and current use of psychoactive medications, lifetime and current diagnoses of alcohol and drug dependence, and current alcohol and drug use. It is important to note that these factors, among other environmental factors, may contribute to further alteration of *MAOA* methylation. Another strength of the study was the use of “gold-standard” genotyping and methylation analyses. Although self-reported measures of maltreatment involve a risk for information bias (Piquero et al. 2014), underreporting often biases reports from official records or other sources (Shaffer et al. 2008). Most participants reported on maltreatment in mid-adolescence and again in the early adulthood, as some participants who were afraid or reluctant to report earlier instances of maltreatment only did so in adulthood. In addition, although group comparisons of methylation levels between abused and non-abused groups were computed using post hoc LSD analyses, similar results were obtained using the more stringent Bonferroni formula to correct alpha levels.

## Conclusions

We show that, in females, SA was associated with hypermethylation of the region spanning the *MAOA* first exon and part of the first intron. This association was robust to adjustments for lifetime and current psychoactive medication, alcohol and drug dependence, and current use of alcohol and drugs. *MAOA* genotype and SA, but not their interactions, were associated with *MAOA* first exon methylation levels. Although SA was directly associated with all mental disorders examined, hypermethylation of the *MAOA* first exon mediated the association between SA and current depression, and was directly associated with lifelong depression. Our results, along with the previous evidence, highlight the importance of epigenetic regulation of this region of *MAOA* for furthering understanding of mechanisms underlying several different mental disorders that appear to depend, to some extent, on type of adversity or trauma experienced.

## Electronic supplementary material

Below is the link to the electronic supplementary material.

Supplementary material 1 (DOCX 187 kb)
