# Supplementary material for: Associations of monoamine oxidase A gene first exon methylation with sexual abuse and current depression in women
Source: J Neural Transm (Vienna). 2018 Mar 29;125(7):1053–64. doi: 10.1007/s00702-018-1875-3 (PMC5999185; doi:10.1007/s00702-018-1875-3)
Supplement: Supplementary file 1 — Supplementary material 1 (DOCX 187 kb) [file 702_2018_1875_MOESM1_ESM.docx]

Online Resource

**Associations of Monoamine Oxidase A Gene First Exon Methylation with Sexual Abuse and Current Depression in Women**

David Checknita, Tomas J. Ekström, Erika Comasco, Kent W. Nilsson, Jari Tiihonen, & Sheilagh Hodgins

**Methods.**

**EpiTYPER Methylation Analysis:** Genomic DNA was first bisulfite-treated using EZ DNA Methylation™ Kit (Zymo Research Corporation, Irvine, California) then assayed using Agena Bioscience’s EpiTYPER at Karolinska University Hospital’s Mutation Analysis Core Facility (MAF). The 448 bp target sequence of *MAOA* region of interest (hg19 chrX: 43,515,544 - 43,515,991) first amplified using a polymerase chain reaction (PCR) with T7-promoter tagged reverse primers to amplify template DNA while retaining bisulfite treatment induced sequence changes. Following shrimp alkaline phosphatase (SAP) treatment and in vitro transcription, uracil-specific enzymatic base cleavage was performed using RNase A. The resulting fragments were assessed using a MALDI-TOF mass-spectrometry technique using the Compact Analyzer intrument to distinguish methylated from unmethylated fragments. Quantitative information from each CpG site within the *MAOA* ROI was generated by the EpiTYPER 1.2 software and used for analyses. Methylation was quantified in duplicate with data retrieved for 14 of the 16 CpGs in the ROI with data for CpGs 1 and 9 not retrievable due to “mass out of range” errors which occur when fragment masses are too low for detection using the technique.

During quality control procedures, to lower the risk of biased amplification of bisulfite-treated unmethylated over methylated DNA, PCR primers were designed to include a number of non-CpG C sites (amplicon designed on the forward strand contained 4, whereas amplicon designed on the reverse strand contained 6 non-CpG C sites). To further assess a possible amplification bias, fully methylated, unmethylated, and a 50% mix of DNA controls (EpiTect, Qiagen, Hilden, Germany) were included in the analysis. No amplification bias was observed in either of the two amplicons. Results of quality control procedures are summarized on Table S6 and Figure S1.

**Results**

**Comparisons of *MAOA* ROI Methylation Levels of participants who had experienced no abuse, one type of abuse, and two types of abuse:** Two-Way Mixed-Model ANOVAs were computed to compare methylation levels of participants who experienced no abuse (n=49), one type of abuse (either PA or SA; n=35), and both types of abuse (both PA and SA; n=30). Results revealed significantly higher levels of overall ROI methylation among those who experienced one type of abuse (p=.002), and two types of abuse (p=.003) as compared to those with no abuse. No differences in overall ROI methylation were detected between those who had experienced one type of abuse and those who had experienced both types of abuse (p=.949). Post-Hoc LSD analysis revealed that those who had experienced one type of abuse showed higher levels of methylation than those who experienced no abuse at CpG sites 2/3 (p=.001), 4 (p<.001), 5/6 (p<.001), 7/8 (p=.001), 10 (p<.001), and 11 (p=.019). Similarly, those who experienced both types of abuse displayed higher levels of methylation than those who experienced no abuse at CpG sites 2/3 (p=.001), 4 (p=.015), 5/6 (p<.001), 7/8 (p=.003), 10 (p<.001), and 11 (p=.003). No differences between those who experienced one or two types of abuse were observed for methylation levels of CpGs 2-11, and no group differences were shown for CpGs 12-16.

**Tables**

**Table S1.** Characteristics of participants carrying different *MAOA* genotypes used as covariates.

|  |  | **MAOA-uVNTR ^a^ (n=114)** | | |  |
| --- | --- | --- | --- | --- | --- |
|  |  | **SS** | **SL** | **LL** | **Statistics** |
| Participants  % Participants |  | n=20  17.5 | n=51  44.7 | n=43  37.7 |  |
| **Psychoactive Medication Use** | | | | | |
| % (n=) Lifetime Psychoactive Medication Use |  | 21.1  (4) | 48.8  (21) | 35.0  (14) | n.s |
| % (n=) Current Psychoactive Medication Use |  | 15.8  (3) | 39.5  (17) | 17.5  (7) | X^2^(2,N=102)=6.54, p=.041 |
| **Alcohol Dependence and Use** | | | | | |
| % (n=) Lifetime Alcohol Dependence |  | 10.0  (2) | 33.3  (17) | 25.6  (11) | n.s |
| % (n=) Current Alcohol Dependence |  | 5.0  (1) | 19.6  (10) | 14.0  (6) | n.s |
| Mean (SD) Current AUDIT Total Score |  | 7.00  (5.13) | 7.39  (5.31) | 7.52  (7.52) | n.s |
| **Drug Dependence and Use** | | | | | |
| % (n=) Lifetime Drug Dependence |  | 25.0  (5) | 23.5  (12) | 30.2  (13) | n.s |
| % (n=) Current Drug Dependence |  | 5.0  (1) | 11.8  (6) | 20.9  (9) | n.s |
| Mean (SD) Current DUDIT Total Score |  | 1.55  (3.70) | 2.39  (5.31) | 3.72  (7.56) | n.s |

| **^a^** Monoamine Oxidase A variable number of tandem repeats.  SD, standard deviation; n.s, not significant, LL, homozygous for the long alleles; SL, heterozygous;  SS, homozygous for the short alleles | | | |
| --- | --- | --- | --- |
|  |  |  |  |
|  |  |  |  |
|  |  |  |  |

**Table S2.** Associations of exclients vs. siblings with lifetime and current clinical diagnoses of alcohol dependence, drug dependence, anxiety disorders, depression disorders, and conduct disorder.

|  |  | **Lifetime Diagnosis^a^** | |  | **Current Diagnosis^b^** | |  |
| --- | --- | --- | --- | --- | --- | --- | --- |
|  |  | **Sexually Abused^c^** | **Non-Abused^d^** | **Statistics** | **Sexually Abused** | **Non-Abused** | **Statistics** |
| Participants |  | n=26 | n=49 |  | n=26 | n=49 |  |
| % Participants |  | 34.7 | 65.3 |  | 34.7 | 65.3 |  |
| % Alcohol dependence |  | 53.8 | 6.1 | X^2^(2,N=75)=22.07  p<.001 | 30.8 | 4.1 | X^2^(2,N=75)=10.47 p=.002 |
| % Drug dependence |  | 50.0 | 8.2 | X^2^(2,N=75)=16.96 p<.001 | 30.8 | 6.1 | X^2^(2,N=75)=8.24  P=.007 |
| % Anxiety Disorders |  | 69.2 | 28.6 | X^2^(2,N=75)=11.48 p=.001 | 53.8 | 24.5 | X^2^(2,N=75)=6.46 p=.021 |
| % Depression Disorders |  | 73.1 | 32.7 | X^2^(2,N=75)=11.15 p=.001 | 42.5 | 24.5 | X^2^(2,N=75)=2.54 p=.124 |
| % Conduct Disorder before age 15 |  | 42.3 | 10.2 | X^2^(2,N=75)=10.43 p=.002 | - | - | - |

^a^ Lifetime diagnosis up to collection of saliva for DNA extraction.

^b^ Current diagnosis at collection of saliva for DNA extraction.

^c^ Participant experienced sexual abuse only.

^d^ Participant did not experience sexual or physical abuse

**Table S3.** Summary of stepwise binary logistic regression models examining *MAOA* exon 1 methylation and SA predictors with lifetime diagnosis of alcohol dependence, drug dependence, anxiety disorders, depressive disorders, and CD before age 15.

|  | **Step 1** | | **Step 2** | | | **Step 3** | |
| --- | --- | --- | --- | --- | --- | --- | --- |
| **Predictor** | **Odds Ratio** | **95% Confidence Interval** | **Odds Ratio** | | **95% Confidence Interval** | **Odds Ratio** | **95% Confidence Interval** |
| Lifetime^a^ Alcohol Dependence | | | | | | | |
| Sexual Abuse | 17.90 | 4.41-72.50 |  | |  | 13.87 | 3.18-60.51 |
| CpG2/3 |  |  | 1.09 | | 1.01-1.18 | 1.03 | 0.94-1.14 |
|  |  |  |  | | | X^2^(1,N=75)=20.92, p<.001 | |
| Sexual Abuse | 17.90 | 4.41-72.50 |  | |  | 17.42 | 3.87-78.37 |
| CpG4 |  |  | 1.07 | | 0.98-1.17 | 0.99 | 0.88-1.11 |
|  |  |  |  | |  | X2(1,N=75)=20.59, p<.001 | |
| Sexual Abuse | 17.90 | 4.41-72.50 |  | |  | 13.37 | 2.98-60.03 |
| CpG5/6 |  |  | 1.12 | | 1.02-1.24 | 1.04 | 0.93-1.17 |
|  |  |  |  | | | X^2^(1,N=75)=20.62, p<.001 | |
| Sexual Abuse | 17.90 | 4.41-72.50 |  | |  | 15.86 | 3.68-68.32 |
| CpG7/8 |  |  | 1.07 | | 0.98-1.16 | 1.01 | 0.92-1.11 |
|  |  |  |  | | | *X*^2^(1,N=75)=20.13, p<.001 | |
| Sexual Abuse | 17.90 | 4.41-72.50 |  | |  | 15.71 | 3.50-70.49 |
| CpG10 |  |  | 1.09 | | 0.99-1.19 | 1.01 | 0.90-1.13 |
|  |  |  |  | | | X^2^(1,N=75)=20.12, p<.001 | |
| Sexual Abuse | 17.90 | 4.41-72.50 |  | |  | 14.68 | 3.22-66.89 |
| Exon 1 |  |  | 1.12 | | 1.01-1.24 | 1.03 | 0.91-1.16 |
|  |  |  |  | |  | X2(1,N=75)=20.24, p<.001 | |
| Sexual Abuse | 17.90 | 4.41-72.50 |  | |  | 18.11 | 4.12-79.60 |
| CpG11 |  |  | 1.05 | | 0.94-1.17 | 0.97 | 0.86-1.10 |
|  |  |  |  | |  | X^2^(1,N=75)=20.24, p<.001 | |
| Sexual Abuse | 17.90 | 4.41-72.50 |  | |  | 26.03 | 5.70-118.91 |
| CpG15 |  |  | 1.02 | | 0.95-1.10 | 1.08 | 0.99-1.17 |
| **S3.** (Continued) | | | | | | | |
|  | Step 1 | | Step 2 | | | Step 3 | |
| **Predictor** | **Odds Ratio** | **95% Confidence Interval** | **Odds Ratio** | | **95% Confidence Interval** | **Odds Ratio** | **95% Confidence Interval** |
|  |  | |  | | |  | |
|  |  |  |  | |  | X^2^(1,N=75)=25.15, p<.001 | |
| Lifetime Drug Dependence | | | | | | | |
| Sexual Abuse | 11.25 | 3.13-40.44 |  | |  | 9.72 | 2.53-37.40 |
| CpG2/3 |  |  | 1.09 | | 1.01-1.18 | 1.05 | 0.95-1.15 |
|  |  |  |  | | | X^2^(1,N=75)=18.38, p<.001 | |
| Sexual Abuse | 11.25 | 3.13-40.44 |  | |  | 10.67 | 2.77-41.15 |
| CpG4 |  |  | 1.10 | | 1.00-1.20 | 1.03 | 0.93-1.15 |
|  |  |  |  | | | X^2^(1,N=75)=17.80 p<.001 | |
| Sexual Abuse | 11.25 | 3.13-40.44 |  | |  | 8.71 | 2.22-34.28 |
| CpG5/6 |  |  | 1.14 | | 1.04-1.25 | 1.07 | 0.96-1.20 |
|  |  |  |  | | | X^2^(1,N=75)=19.04, p<.001 | |
| Sexual Abuse | 11.25 | 3.13-40.44 |  | |  | 11.19 | 2.94-42.65 |
| CpG7/8 |  |  | 1.07 | | 0.99-1.16 | 1.02 | 0.93-1.12 |
|  |  |  |  | | | *X*^2^(1,N=75)=17.63, p<.001 | |
| Sexual Abuse | 11.25 | 3.13-40.44 |  | |  | 9.40 | 2.46-35.96 |
| CpG10 |  |  | 1.12 | | 1.02-1.24 | 1.07 | 0.96-1.19 |
|  |  |  |  | | | X^2^(1,N=75)=18.87, p<.001 | |
| Sexual Abuse | 11.25 | 3.13-40.44 |  | |  | 9.21 | 2.33-36.41 |
| Exon 1 |  |  | 1.14 | | 1.03-1.26 | 1.06 | 0.95-1.20 |
|  |  |  |  | | | X^2^(1,N=75)=18.52, p<.001 | |
| Sexual Abuse | 11.25 | 3.13-40.44 |  |  | | 10.42 | 2.79-38.88 |
| CpG11 |  |  | 1.12 | 1.00-1.25 | | 1.07 | 0.95-1.20 |
|  |  |  |  |  | | X^2^(1,N=75)=18.59, p<.001 | |
| Sexual Abuse | 11.25 | 3.13-40.44 |  |  | | 13.62 | 3.55-52.25 |
| CpG15 |  |  | 1.00 | 0.94-1.08 | | 1.04 | 0.97-1.13 |
|  |  |  |  |  | | X^2^(1,N=75)=17.82, p<.001 | |
|  |  |  |  |  | |  | |
| **S3.** (Continued) |  | |  | | |  | |
|  | **Step 1** | | **Step 2** | | | **Step 3** | |
| **Predictor** | **Odds Ratio** | **95% Confidence Interval** | **Odds Ratio** | | **95% Confidence Interval** | **Odds Ratio** | **95% Confidence Interval** |
| Lifetime Anxiety Disorders | | | | | | | |
| Sexual Abuse | 5.63 | 1.99-15.88 |  | |  | 3.99 | 1.32-12.01 |
| CpG2/3 |  |  | 1.09 | | 1.02-1.17 | 1.06 | 0.98-1.14 |
|  |  |  |  | | | X^2^(1,N=75)=13.00, p=.002 | |
| Sexual Abuse | 5.63 | 1.99-15.88 |  | |  | 4.89 | 1.62-14.77 |
| CpG4 |  |  | 1.06 | | 0.98-1.06 | 1.02 | 0.93-1.12 |
|  |  |  |  | |  | X^2^(1,N=75)=10.84, p=.004 | |
| Sexual Abuse | 5.63 | 1.99-15.88 |  | |  | 3.98 | 1.13-12.27 |
| CpG5/6 |  |  | 1.10 | | 1.02-1.19 | 1.06 | 0.97-1.16 |
|  |  |  |  | | | X^2^(1,N=75)=12.28, p=.002 | |
| Sexual Abuse | 5.63 | 1.99-15.88 |  | |  | 4.58 | 1.55-13.52 |
| CpG7/8 |  |  | 1.07 | | 0.99-1.15 | 1.04 | 0.96-1.13 |
|  |  |  |  | | | X^2^(1,N=75)=11.56, p=.003 | |
| Sexual Abuse | 5.63 | 1.99-15.88 |  | |  | 3.74 | 1.23-11.34 |
| CpG10 |  |  | 1.13 | | 1.04-1.23 | 1.09 | 1.00-1.19 |
|  |  |  |  | | | X^2^(1,N=75)=14.70, p=.001 | |
| Sexual Abuse | 5.63 | 1.99-15.88 |  | |  | 3.82 | 1.24-11.77 |
| Exon 1 |  |  | 1.13 | | 1.03-1.23 | 1.08 | 0.98-1.19 |
|  |  |  |  | | | X^2^(1,N=75)=12.87, p=.002 | |
| Sexual Abuse | 5.63 | 1.99-15.88 |  |  | | 4.36 | 1.48-12.82 |
| CpG11 |  |  | 1.12 | 1.02-1.24 | | 1.09 | 0.99-1.21 |
|  |  |  |  |  | | X^2^(1,N=75)=13.95, p=.001 | |
| Sexual Abuse | 5.63 | 1.99-15.88 |  |  | | 6.76 | 2.22-20.55 |
| CpG15 |  |  | 1.01 | 0.95-1.07 | | 1.04 | 0.97-1.11 |
|  |  |  |  |  | | X^2^(1,N=75)=13.00, p=.002 | |
|  |  |  |  |  | |  | |
|  |  |  |  |  | |  | |
|  |  |  |  |  | |  | |
| **S3.** (Continued) |  | |  | | |  | |
|  | **Step 1** | | **Step 2** | | | **Step 3** | |
| **Predictor** | **Odds Ratio** | **95% Confidence Interval** | **Odds Ratio** | | **95% Confidence Interval** | **Odds Ratio** | **95% Confidence Interval** |
| Lifetime Depression Disorders | | | | | | | |
| Sexual Abuse | 5.60 | 1.95-16.04 |  | |  | 3.75 | 1.13-11.50 |
| CpG2/3 |  |  | 1.10 | | 1.03-1.19 | 1.07 | 1.00-1.60 |
|  |  |  |  | | | X^2^(1,N=75)=14.21, p=.001 | |
| Sexual Abuse | 5.60 | 1.95-16.04 |  | |  | 4.83 | 1.58-14.75 |
| CpG4 |  |  | 1.07 | | 0.98-1.15 | 1.02 | 0.93-1.12 |
|  |  |  |  | |  | X^2^(1,N=75)=10.78, p=.005 | |
| Sexual Abuse | 5.60 | 1.95-16.04 |  | |  | 3.37 | 1.08-10.54 |
| CpG5/6 |  |  | 1.14 | | 1.05-1.24 | 1.10 | 1.01-1.21 |
|  |  |  |  | | | X^2^(1,N=75)=15.30, p<.001 | |
| Sexual Abuse | 5.60 | 1.95-16.04 |  | |  | 4.21 | 1.41-12.59 |
| CpG7/8 |  |  | 1.10 | | 1.02-1.19 | 1.07 | 0.99-1.17 |
|  |  |  |  | | | X^2^(1,N=75)=13.36, p=.001 | |
| Sexual Abuse | 5.60 | 1.95-16.04 |  | |  | 4.33 | 1.42-13.21 |
| CpG10 |  |  | 1.09 | | 1.00-1.18 | 1.05 | 0.96-1.14 |
|  |  |  |  | | | X^2^(1,N=75)=11.68, p=.003 | |
| Sexual Abuse | 5.60 | 1.95-16.04 |  | |  | 3.63 | 1.16-11.31 |
| Exon 1 |  |  | 1.14 | | 1.04-1.25 | 1.09 | 0.99-1.21 |
|  |  |  |  | |  | X^2^(1,N=75)=13.03, p=.001 | |
| Sexual Abuse | 5.60 | 1.95-16.04 |  | |  | 4.53 | 1.53-13.40 |
| CpG11 |  |  | 1.10 | | 1.00-1.20 | 1.07 | 0.97-1.18 |
|  |  |  |  | |  | X^2^(1,N=75)=12.32, p=.002 | |
| Sexual Abuse | 5.60 | 1.95-16.04 |  | |  | 7.08 | 2.26-22.19 |
| CpG15 |  |  | 1.01 | | 0.96-1.07 | 1.05 | 0.98-1.12 |
|  |  |  |  | |  | X^2^(1,N=75)=13.44, p=.001 | |
|  |  |  |  | |  |  | |
|  |  |  |  | |  |  |  |
|  |  |  |  | |  |  |  |
| **S3.** (Continued) |  | |  | | |  | |
|  | **Step 1** | | **Step 2** | | | **Step 3** | |
| **Predictor** | **Odds Ratio** | **95% Confidence Interval** | **Odds Ratio** | | **Predictor** | **Odds Ratio** | **95% Confidence Interval** |
| Conduct Disorder before age 15 | | | | | | | |
| Sexual Abuse | 6.45 | 1.93-21.61 |  | |  | 5.05 | 1.41-18.11 |
| CpG2/3 |  |  | 1.10 | | 1.02-1.19 | 1.07 | 1.07-1.16 |
|  |  |  |  | | | X^2^(1,N=75)=12.97, p=.002 | |
| Sexual Abuse | 6.45 | 1.93-21.61 |  | |  | 8.26 | 2.17-31.41 |
| CpG4 |  |  | 1.03 | | 0.94-1.13 | 0.96 | 0.87-1.07 |
|  |  |  |  | |  | X^2^(1,N=75)=11.14, p=.004 | |
| Sexual Abuse | 6.45 | 1.93-21.61 |  | |  | 5.17 | 1.38-19.31 |
| CpG5/6 |  |  | 1.11 | | 1.01-1.22 | 1.06 | 0.95-1.17 |
|  |  |  |  | | | X^2^(1,N=75)=11.79, p=.003 | |
| Sexual Abuse | 6.45 | 1.93-21.61 |  | |  | 5.30 | 1.15-18.75 |
| CpG7/8 |  |  | 1.11 | | 1.02-1.21 | 1.08 | 0.98-1.18 |
|  |  |  |  | | | X^2^(1,N=75)=13.28, p=.001 | |
| Sexual Abuse | 6.45 | 1.93-21.61 |  | |  | 6.93 | 1.84-26.06 |
| CpG10 |  |  | 1.06 | | 0.97-1.16 | 0.99 | 0.90-1.11 |
|  |  |  |  | | | X^2^(1,N=75)=10.68, p=.005 | |
| Sexual Abuse | 6.45 | 1.93-21.61 |  | |  | 5.42 | 1.45-20.28 |
| Exon 1 |  |  | 1.11 | | 1.01-1.23 | 1.05 | 0.94-1.18 |
|  |  |  |  | | | X^2^(1,N=75)=11.45, p=.003 | |
| Sexual Abuse | 6.45 | 1.93-21.61 |  |  | | 5.99 | 1.71-20.93 |
| CpG11 |  |  | 1.10 | 0.99-1.23 | | 1.06 | 0.94-1.19 |
|  |  |  |  |  | | X^2^(1,N=75)=11.52, p=.003 | |
| Sexual Abuse | 6.45 | 1.93-21.61 |  |  | | 6.15 | 1.78-21.17 |
| CpG15 |  |  | 0.96 | 0.90-1.03 | | 0.99 | 0.92-1.06 |
|  |  |  |  |  | | X^2^(1,N=75)=10.16, p=.006 | |
|  |  |  |  |  | |  |  |
|  |  |  |  |  | |  |  |

**Table S4.** Summary of stepwise binary logistic regression models examining *MAOA* exon 1 methylation and SA predictors with current diagnosis of alcohol dependence, drug dependence, anxiety disorders, and depressive disorders.

|  | **Step 1** | | **Step 2** | | | **Step 3** | |
| --- | --- | --- | --- | --- | --- | --- | --- |
| **Predictor** | **Odds Ratio** | **95% Confidence Interval** | **Odds Ratio** | | **95% Confidence Interval** | **Odds Ratio** | **95% Confidence Interval** |
| Current^a^ Alcohol Dependence | | | | | | | |
| Sexual Abuse | 10.44 | 2.02-53.95 |  | |  | 6.92 | 1.21-39.42 |
| CpG2/3 |  |  | 1.10 | | 1.00-1.20 | 1.05 | 0.95-1.18 |
|  |  |  |  | | | X^2^(1,N=75)=9.33, p=.009 | |
| Sexual Abuse | 10.44 | 2.02-53.95 |  | |  | 8.80 | 1.52-50.82 |
| CpG4 |  |  | 1.07 | | 0.95-1.20 | 1.01 | 0.89-1.14 |
|  |  |  |  | |  | X2(1,N=75)=8.44, p=.015 | |
| Sexual Abuse | 10.44 | 2.02-53.95 |  | |  | 5.11 | 0.86-30.25 |
| CpG5/6 |  |  | 1.18 | | 1.04-1.34 | 1.13 | 0.93-1.29 |
|  |  |  |  | | | X^2^(1,N=75)=11.58, p=.003 | |
| Sexual Abuse | 10.44 | 2.02-53.95 |  | |  | 7.59 | 1.36-42.41 |
| CpG7/8 |  |  | 1.08 | | 0.99-1.19 | 1.04 | 0.94-1.16 |
|  |  |  |  | | | *X*^2^(1,N=75)=9.10, p=.011 | |
| Sexual Abuse | 10.44 | 2.02-53.95 |  | |  | 6.57 | 1.16-37.21 |
| CpG10 |  |  | 1.13 | | 1.00-1.27 | 1.08 | 0.95-1.23 |
|  |  |  |  | | | X^2^(1,N=75)=9.82, p=.007 | |
| Sexual Abuse | 10.44 | 2.02-53.95 |  | |  | 6.21 | 1.05-36.87 |
| Exon 1 |  |  | 1.15 | | 1.01-1.31 | 1.08 | 0.94-1.25 |
|  |  |  |  | |  | X2(1,N=75)=9.71, p=.008 | |
| Sexual Abuse | 10.44 | 2.02-53.95 |  | |  | 9.37 | 1.68-52.34 |
| CpG11 |  |  | 1.05 | | 0.92-1.20 | 0.99 | 0.86-1.14 |
|  |  |  |  | |  | X^2^(1,N=75)=8.43, p=.015 | |
| Sexual Abuse | 10.44 | 2.02-53.95 |  | |  | 11.80 | 2.20-63.39 |
| CpG15 |  |  | 1.00 | | 0.92-1.09 | 1.03 | 0.95-1.13 |
|  |  |  |  | |  |  |  |
|  |  |  |  | |  |  |  |
| **S4.** (Continued) | | | | | | | |
|  | **Step 1** | | **Step 2** | | | **Step 3** | |
| **Predictor** | **Odds Ratio** | **95% Confidence Interval** | **Odds Ratio** | | **95% Confidence Interval** | **Odds Ratio** | **95% Confidence Interval** |
|  |  | |  | | |  | |
|  |  |  |  | |  | X^2^(1,N=75)=10.63, p=.005 | |
| Current Drug Dependence | | | | | | | |
| Sexual Abuse | 6.81 | 1.62-28.60 |  | |  | 6.80 | 1.44-32.05 |
| CpG2/3 |  |  | 1.06 | | 0.97-1.15 | 1.01 | 0.91-1.12 |
|  |  |  |  | | | X^2^(1,N=75)=8.34, p=.015 | |
| Sexual Abuse | 6.81 | 1.62-28.60 |  | |  | 6.60 | 1.44-30.25 |
| CpG4 |  |  | 1.07 | | 0.97-1.19 | 1.02 | 0.91-1.15 |
|  |  |  |  | | | X^2^(1,N=75)=8.41 p=.015 | |
| Sexual Abuse | 6.81 | 1.62-28.60 |  | |  | 5.27 | 1.11-25.05 |
| CpG5/6 |  |  | 1.12 | | 1.00-1.24 | 1.06 | 0.94-1.19 |
|  |  |  |  | | | X^2^(1,N=75)=9.26, p=.010 | |
| Sexual Abuse | 6.81 | 1.62-28.60 |  | |  | 6.83 | 1.52-30.67 |
| CpG7/8 |  |  | 1.05 | | 0.96-1.15 | 1.01 | 0.92-1.12 |
|  |  |  |  | | | *X*^2^(1,N=75)=8.36, p=.015 | |
| Sexual Abuse | 6.81 | 1.62-28.60 |  | |  | 7.74 | 1.16-37.21 |
| CpG10 |  |  | 1.05 | | 0.95-1.16 | 0.99 | 0.87-1.11 |
|  |  |  |  | | | X^2^(1,N=75)=8.35, p=.015 | |
| Sexual Abuse | 6.81 | 1.62-28.60 |  | |  | 6.40 | 1.33-30.87 |
| Exon 1 |  |  | 1.09 | | 0.98-1.22 | 1.02 | 0.90-1.17 |
|  |  |  |  | | | X^2^(1,N=75)=8.43, p=.015 | |
| Sexual Abuse | 6.81 | 1.62-28.60 |  |  | | 5.67 | 1.28-25.07 |
| CpG11 |  |  | 1.15 | 1.00-1.31 | | 1.10 | 0.95-1.26 |
|  |  |  |  |  | | X^2^(1,N=75)=10.14, p=.006 | |
| Sexual Abuse | 6.81 | 1.62-28.60 |  |  | | 6.92 | 1.60-30.01 |
| CpG15 |  |  | 0.98 | 0.91-1.06 | | 1.00 | 0.93-1.09 |
|  |  |  |  |  | | X^2^(1,N=75)=7.87, p=.019 | |
|  |  |  |  |  | |  | |
| **S4.** (Continued) |  | |  | | |  | |
|  | **Step 1** | | **Step 2** | | | **Step 3** | |
| **Predictor** | **Odds Ratio** | **95% Confidence Interval** | **Odds Ratio** | | **95% Confidence Interval** | **Odds Ratio** | **95% Confidence Interval** |
| Current Anxiety Disorders | | | | | | | |
| Sexual Abuse | 3.60 | 1.31-9.87 |  | |  | 3.52 | 1.17-10.57 |
| CpG2/3 |  |  | 1.05 | | 0.98-1.12 | 1.02 | 0.95-1.10 |
|  |  |  |  | | | X^2^(1,N=75)=7.35, p=.025 | |
| Sexual Abuse | 3.60 | 1.31-9.87 |  | |  | 3.52 | 1.19-10.43 |
| CpG4 |  |  | 1.06 | | 0.98-1.15 | 1.03 | 0.94-1.12 |
|  |  |  |  | |  | X^2^(1,N=75)=7.41, p=.025 | |
| Sexual Abuse | 3.60 | 1.31-9.87 |  | |  | 3.23 | 1.05-9.90 |
| CpG5/6 |  |  | 1.07 | | 0.99-1.16 | 1.04 | 0.95-1.13 |
|  |  |  |  | | | X^2^(1,N=75)=7.74, p=.021 | |
| Sexual Abuse | 3.60 | 1.31-9.87 |  | |  | 3.52 | 1.21-10.28 |
| CpG7/8 |  |  | 1.05 | | 0.98-1.13 | 1.03 | 0.95-1.11 |
|  |  |  |  | | | X^2^(1,N=75)=7.51, p=.023 | |
| Sexual Abuse | 3.60 | 1.31-9.87 |  | |  | 3.29 | 1.10-9.80 |
| CpG10 |  |  | 1.07 | | 0.99-1.16 | 1.04 | 0.95-1.13 |
|  |  |  |  | | | X^2^(1,N=75)=7.86, p=.020 | |
| Sexual Abuse | 3.60 | 1.31-9.87 |  | |  | 3.23 | 1.05-9.91 |
| Exon 1 |  |  | 1.08 | | 0.99-1.18 | 1.04 | 0.94-1.15 |
|  |  |  |  | | | X^2^(1,N=75)=7.74, p=.021 | |
| Sexual Abuse | 3.60 | 1.31-9.87 |  |  | | 3.34 | 1.16-9.62 |
| CpG11 |  |  | 1.09 | 0.99-1.20 | | 1.06 | 0.96-1.17 |
|  |  |  |  |  | | X^2^(1,N=75)=8.60, p=.014 | |
| Sexual Abuse | 3.60 | 1.31-9.87 |  |  | | 3.74 | 1.32-10.61 |
| CpG15 |  |  | 0.99 | 0.93-1.05 | | 1.01 | 0.95-1.07 |
|  |  |  |  |  | | X^2^(1,N=75)=6.46, p=.040 | |
|  |  |  |  |  | |  | |
|  |  |  |  |  | |  |  |
|  |  |  |  |  | |  |  |
| **S4.** (Continued) |  | |  | | |  | |
|  | **Step 1** | | **Step 2** | | | **Step 3** | |
| **Predictor** | **Odds Ratio** | **95% Confidence Interval** | **Odds Ratio** | | **95% Confidence Interval** | **Odds Ratio** | **95% Confidence Interval** |
| Current Depression Disorders | | | | | | | |
| Sexual Abuse | 5.60 | 1.95-16.04 |  | |  | 1.20 | 0.38-3.80 |
| CpG2/3 |  |  | 1.11 | | 1.03-1.20 | 1,11 | 1.02-1.20 |
|  |  |  |  | | | X^2^(1,N=75)=8.77, p=.012 | |
| Sexual Abuse | 5.60 | 1.95-16.04 |  | |  | 1.38 | 0.44-4.26 |
| CpG4 |  |  | 1.11 | | 1.02-1.21 | 1.10 | 1.00-1.21 |
|  |  |  |  | |  | X^2^(1,N=75)=6.05, p=.049 | |
| Sexual Abuse | 5.60 | 1.95-16.04 |  | |  | 1.01 | 0.30-3.35 |
| CpG5/6 |  |  | 1.14 | | 1.04-1.25 | 1.14 | 1.04-1.26 |
|  |  |  |  | | | X^2^(1,N=75)=9.96, p=.007 | |
| Sexual Abuse | 5.60 | 1.95-16.04 |  | |  | 1.40 | 0.46-4.29 |
| CpG7/8 |  |  | 1.11 | | 1.02-1.20 | 1.10 | 1.01-1.19 |
|  |  |  |  | | | X^2^(1,N=75)=7.10, p=.029 | |
| Sexual Abuse | 5.60 | 1.95-16.04 |  | |  | 1.38 | 0.45-4.27 |
| CpG10 |  |  | 1.10 | | 1.01-1.20 | 1.09 | 1.00-1.19 |
|  |  |  |  | | | X^2^(1,N=75)=5.55, p=.062 | |
| Sexual Abuse | 5.60 | 1.95-16.04 |  | |  | 1.02 | 0.31-3.39 |
| Exon 1 |  |  | 1.16 | | 1.05-1.28 | 1.16 | 1.04-1.29 |
|  |  |  |  | |  | X^2^(1,N=75)=9.74, p=.008 | |
| Sexual Abuse | 5.60 | 1.95-16.04 |  | |  | 1.59 | 0.53-4.73 |
| CpG11 |  |  | 1.11 | | 1.00-1.23 | 1.09 | 0.99-1.21 |
|  |  |  |  | |  | X^2^(1,N=75)=5.00, p=.082 | |
| Sexual Abuse | 5.60 | 1.95-16.04 |  | |  | 2.37 | 0.83-6.75 |
| CpG15 |  |  | 1.00 | | 0.94-1.06 | 1.01 | 0.95-1.08 |
|  |  |  |  | |  | X^2^(1,N=75)=2.62, p=.270 | |
|  |  |  |  | |  |  |  |

**Table S5.** Summary of predicted transcription factor binding domains in MAOA exon 1 as indicated by Transfac.

| **CpG #** | **Transcription Factor Names** | **Enhancer/Repressor** |
| --- | --- | --- |
| 2/3 | RXR-alpha, GR-alpha, C-Jun | Enhancer (All) |
| 4 | GATA1 | Enhancer |
| 5/6 | GATA1 | Enhancer |
| 7/8 | PR-A | Enhancer |
| 10 | GR-alpha | Enhancer |

**Table S6.** Methylation levels of the positive controls at each *MAOA* ROI CpG site.

| **Positive controls** | | **CPG site within the ROI** | | | | | | | | | | | | | |
| --- | --- | --- | --- | --- | --- | --- | --- | --- | --- | --- | --- | --- | --- | --- | --- |
|  |  | **1^#^** | **2 & 3** | **4** | **5 & 6** | **7 & 8** | **9^#^** | **10** | **11** | **12** | **13** | **14** | **15** | **16** |  |
|  |  | **UCSC position (chrX, hg19)** | | | | | | | | | | | | | |
|  |  | 43515544 | 43515575 &  43515564 | 43515609 | 43515618 &  43515616 | 43515634 &  43515631 | 43515640 | 43515646 | 43515675 | 43515680 | 43515762 | 43515801 | 43515936 | 43515990 |  |
| 0 %  Methylated  control | 1 | NA | 0.02 | 0.09 | 0.05 | 0.01 | NA | 0.04 | 0.05 | 0.02 | 0.03 | 0.03 | 0.09 | 0.07 |  |
|  | 2 | NA | 0.03 | 0.07 | 0.04 | 0.04 | NA | 0.01 | 0.08 | 0.19 | 0.05 | 0.00 | 0.06 | 0.09 |  |
|  | 3 | NA | 0.06 | 0.09 | 0.04 | 0.01 | NA | 0.01 | 0.04 | 0.09 | 0.03 | 0.00 | 0.07 | 0.03 |  |
|  | 4 | NA | 0.03 | 0.12 | 0.05 | 0.03 | NA | 0.08 | 0.03 | 0.19 | 0.04 | 0.05 | 0.06 | 0.03 |  |
|  | 5 | NA | 0.04 | 0.08 | 0.07 | 0.04 | NA | 0.00 | 0.06 | 0.15 | 0.07 | 0.02 | 0.06 | 0.07 |  |
| 50 % methylated  control | 1 | NA | 0.73 | 0.70 | 0.73 | 0.62 | NA | 0.64 | 0.72 | 0.70 | 0.70 | 0.65 | 0.74 | 0.70 |  |
|  | 2 | NA | 0.69 | 0.68 | 0.67 | 0.53 | NA | 0.57 | 0.66 | 0.61 | 0.62 | 0.47 | 0.51 | 0.51 |  |
|  | 3 | NA | 0.64 | 0.57 | 0.58 | 0.57 | NA | 0.53 | 0.57 | 0.66 | 0.60 | 0.74 | 0.77 | 0.77 |  |
|  | 4 | NA | 0.67 | 0.67 | 0.66 | 0.64 | NA | 0.54 | 0.67 | 0.67 | 0.59 | 0.63 | 0.40 | 0.68 |  |
|  | 5 | NA | 0.55 | 0.56 | 0.54 | 0.51 | NA | 0.44 | 0.55 | 0.55 | 0.53 | 0.55 | 0.59 | 0.61 |  |
| 100% methylated  control | 1 | NA | 0.90 | 0.91 | 0.92 | 0.88 | NA | 1.00 | 0.99 | 0.98 | 0.96 | 0.85 | 0.97 | 0.94 |  |
|  | 2 | NA | 0.90 | 0.93 | 0.93 | 0.83 | NA | 0.81 | 0.95 | 0.95 | 0.98 | 0.90 | 0.96 | 0.97 |  |
|  | 3 | NA | 0.87 | 0.86 | 0.91 | 0.83 | NA | 0.84 | 0.99 | 0.94 | 0.94 | 0.80 | 0.99 | 0.95 |  |
|  | 4 | NA | 0.87 | 0.87 | 0.91 | 0.86 | NA | 0.84 | 0.95 | 0.94 | 0.94 | 0.91 | 0.95 | 0.96 |  |
|  | 5 | NA | 0.91 | 0.89 | 0.93 | 0.90 | NA | 0.86 | 1.00 | 0.97 | 0.93 | 0.90 | 0.98 | 0.94 |  |

**Figures**

**Figure S1.** Methylation levels of positive controls measured at each *MAOA* ROI CpG site.

**
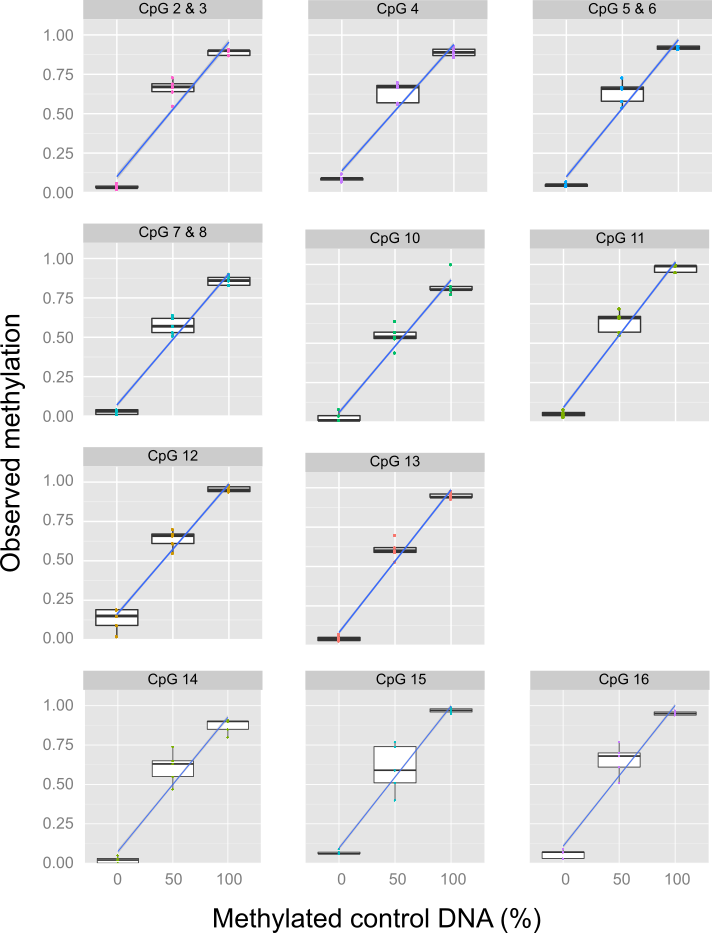
**

Positive controls were fully-methylated (100%, n = 5), semi-methylated (50%, n= 5) and un-methylated (0%, n = 5). Methylation values for positive controls at each locus are shown on Table S7.
